# Effects of a Nine-Month Physical Activity Intervention on Morphological Characteristics and Motor and Cognitive Skills of Preschool Children

**DOI:** 10.3390/ijerph17186609

**Published:** 2020-09-11

**Authors:** Damjan Jaksic, Sandra Mandic, Nebojsa Maksimovic, Zoran Milosevic, Roberto Roklicer, Jovan Vukovic, Suncica Pocek, Nemanja Lakicevic, Antonino Bianco, Samuel Cassar, Patrik Drid

**Affiliations:** 1 Faculty of Sport and Physical Education, University of Novi Sad, 21000 Novi Sad, Serbia; jaksic_damjan@yahoo.com (D.J.); nebojsam@uns.ac.rs (N.M.); zoranaisns29@gmail.com (Z.M.); roklicer.r@gmail.com (R.R.); jovanvukovic89@gmail.com (J.V.); suncicapocekfsfv@gmail.com (S.P.); 2 Active Living Laboratory, School for Physical Education, Sport and Exercise Sciences, University of Otago, Dunedin 9016, New Zealand; sandy07a@gmail.com; 3 PhD Program in Health Promotion and Cognitive Sciences, University of Palermo, 90100 Palermo, Italy; lakinem89@gmail.com; 4 Sport and Exercise Science Research Unit, University of Palermo, 90100 Palermo, Italy; antonino.bianco@unipa.it; 5 Institute for Physical Activity and Nutrition (IPAN), School of Exercise and Nutrition Sciences, Deakin University, Geelong 3216, Australia; scas@deakin.edu.au

**Keywords:** motor skills, cognitive function, physical development, physical activity

## Abstract

(1) Background: Regular physical activity (PA) plays an important role during early childhood physical and psychological development. This study investigates the effects of a 9-month PA intervention on physiological characteristics and motor and cognitive skills in preschool children. (2) Methods: Preschool children (*n* = 132; age 4 to 7 years) attending regular preschool programs were nonrandomly assigned to PA intervention (*n* = 66; 60 min sessions twice per week) or a control group (*n* = 66; no additional organized PA program) for 9 months. Exercise training for the intervention group included various sports games, outdoor activities, martial arts, yoga, and dance. Anthropometry, motor skills (7 tests), and cognitive skills (Raven’s Colored Progressive Matrices and Cognitive Assessment System) were assessed before and after an intervention period in both groups. Data were analyzed using repeated-measures ANOVA. (3) Results: Body weight significantly increased in both groups over time. Compared to the changes observed in the control group, the intervention group significantly increased in chest circumference (*p* = 0.022). In contrast, the control group demonstrated an increase in waist circumference (*p* = 0.001), while these measures in the intervention group remained stable. Participants in the intervention group improved running speed (*p* = 0.016) and standing broad jump (*p* = 0.000). The flexibility level was maintained in the intervention group, while a significant decrease was observed in the control group (*p* = 0.010). Children from the intervention group demonstrated progress in the bent-arm hang test (*p* = 0.001), unlike the control group subjects. Varied improvements in cognitive skills were observed for different variables in both intervention and control groups, with no robust evidence for PA-intervention-related improvements. (4) Conclusions: Preschool children’s participation in a preschool PA intervention improves their motor skills.

## 1. Introduction

Early childhood represents a crucial period for children’s physical and psychological development [1]. Regular physical activity (PA) during this time frame has numerous health benefits [2,3]. Regular PA in childhood does not only provide healthy body weight but also contributes to a vast array of physiological and psychosocial benefits [4,5]. Despite numerous health benefits of PA during early childhood [2,3], PA levels in preschool children remain relatively low [6]. Reasons for this phenomenon range from environmental to individual [7]. A study on 1004 preschool children in Australia showed that only 16% of children spent a substantial amount of time engaging in daily PA [8]. Furthermore, Tremblay et al. [9] reported that only 7% of Canadian children and youth participate in at least one hour of moderate- to vigorous-intensity PA per day, thus meeting the current PA recommendations from World Health Organization [10]. However, it is very important to know that even if the criteria of one hour per day of PA is fulfilled by children, there is still another 23 h in a day for other activities, school, rest, and free time. Studies have repeatedly shown that children and youth spend the majority of their leisure time engaging in sedentary pursuits [9,11]. Considering the accelerated physical development in early childhood (up to 5 years of age) [12], parents and educators should emphasize the development of basic motor skills such as running, jumping, throwing, catching, and kicking during this period. Children with better motor skills are likely to engage in a particular sport or leisure-time physical activity (PA) [13]. The necessity of developing basic motor skills will also increase the likelihood of participating in regular PA across the lifespan [14].

According to the current PA guidelines for youth, put together by the American College of Sports Medicine, children should accumulate at least one hour of PA daily as part of commuting, physical education, sport, free play, and structured exercise, whereby moderate- to vigorous-intensity of PA (MVPA) should be emphasized [15]. PA recommendations for preschool children differ markedly from school-age children and adolescents. Preschoolers’ PA is primarily defined as play, and their activity habits tend to be sporadic, varying significantly in intensity [16]. Bearing the abovementioned in mind, PA interventions for preschoolers should focus on the development of fundamental motor skill competence through play [17], which can facilitate children’s participation in sports. Indeed, developing motor skills in preschool children is of the utmost importance for their healthy development and obesity prevention [18]. However, emerging data differentiate PA recommendations for children 3–4 years old and children beyond 5 years of age. Preschool children (aged 3–4 years) should accumulate at least 180 min of PA and engage in no more than 1 h sedentary screen time, whereas school-age children and adolescents (5–17 years) should aim for at least 60 min of MVPA and engage in no more than 2 h sedentary recreational screen time [19]. Despite differentiating the amount of PA recommended for preschool and school-age children, the latter recommendations also distinguish the amount of sedentary pursuits children should engage in, making it more comprehensive and concise regarding light PA (LPA), moderate-to-vigorous PA, and sedentary behavior [19].

Recent studies have shown that participation in PA may modify white matter integrity and enhance cognitive processes in youth [20,21,22]. Moreover, a myriad of studies has reported that sedentary behavior might be a risk factor contributing to an early onset of cognitive decline in adults [23]. Previous research has also shown that the effects of PA on cognition also depend on the type of cognitive task performed and the PA duration [24]. PA affects the improvement of cognitive control [25,26] along with the development of attention span [27,28]. Emerging research in neuroscience has discovered a tangible relationship between PA and cognitive performance as well as brain structure and function [29]. A meta-analysis that included 33,816 participants showed that subjects who performed a high level of PA were significantly protected (−38%) against cognitive decline [23]. Moreover, accumulating evidence shows that regular PA has positive effects on structural and functional aspects of the brain (e.g., increased neurotrophins, cerebral blood flow, or grey matter volume), which leads to enhanced cognitive function [30]. Therefore, the aim of this study was to examine the effects of a 9-month PA intervention versus a standard preschool activity program in preschool institutions on motor skills and cognitive performance in children aged 4 to 7 years.

## 2. Materials and Methods

### 2.1. Subjects

A total of 132 children (boys = 72, girls = 60; age range 4–7 years) participated in this study for a nine-month period. All children attended a standard preschool program during the day at one of the preschool institutions in Novi Sad, Serbia. The intervention group consisted of 66 children who attended the preschool PA program (two 60-min sessions per week) in addition to their participation in a standard preschool institution program. The control group consisted of 66 healthy children from a single preschool institution who did not engage in any additional organized PA outside of the standard preschool program. All participants’ parents gave written consent for their children to participate in the study. This study was approved by the Institutional Review Committee of the University of Novi Sad (Ref. No. 44-01-02/2018-1) and was conducted under the Declaration of Helsinki.

### 2.2. Experimental Design

All participants were tested before and after the 9-month intervention period. The intervention group attended the preschool PA program (two 60-min sessions per week; an intervention) in addition to their participation in a standard preschool institution program. The control group participated in the standard preschool program but did not engage in any additional organized PA outside of that program. Preintervention and postintervention assessments in both groups included measurements of anthropometric characteristics, motor skills, and cognitive function (Raven’s Coloured Progressive Matrices and Cognitive Assessment System (CAS).

### 2.3. Physical Activity Intervention

The intervention group participated in a PA intervention that consisted of two 60-min PA sessions weekly over a period of 9 months (September 2018 to May 2019). PA intervention was carried out by experienced physical education teachers, specialists who work with preschool children. The intervention included the following activities: athletics, sports games (football, basketball, handball, volleyball, and tennis), gymnastics, yoga, outdoor activities, aerobic, martial arts, and dance (Table 1). No significant gender differences were observed in terms of anthropometric characteristics, motor skills, and cognitive abilities within the experimental group at the initial measurements.

The skill-related component of the PA intervention included balance, coordination, timing, agility, balance, speed, flexibility, strength, endurance, and agility-based activities. One of the goals of this component of the intervention was to improve the speed of solving complex motor problems, which is a part of cognitive training. Types of exercises included perceptual-motor activities, creative movements, rhythm and dance, stunts, tumbling, running, jumping, throwing, games, and basic elements of team sports. This variety of movement raises the interest of preschool children in skill-related training. The control group was exposed to the standard program for preschool institutions.

### 2.4. Anthropometric Characteristics

Anthropometric characteristics were assessed according to the recommendations of the International Biological Program (IBP) [31] and included the following: height, weight, chest circumference, waist circumference, forearm circumference, abdominal skinfold, subscapular skinfold, and triceps skinfold. Body height was measured to the nearest 0.5 cm using a Martin metal anthropometer (GPM Switzerland). Body weight was measured to the nearest 0.1 kg using an Omron weight scale BF511 (Omron, Osaka, Japan). Chest, waist, and forearm circumferences were determined by centimetric tape to 0.1 cm. Skinfold thickness was measured using a John Bull caliper (British Indicator Ltd., Thornaby, UK), accurate to 0.1 mm. All anthropometric measurements were completed by experienced and well-trained persons.

### 2.5. Motor Skills Assessment

The assessment of motor skills included seven tests that are described in detail below [32,33].

**20-m dash**. Participants stood behind the start line until the command “GO”, after which they had to run for 20 m as fast as possible. Participants were instructed to run in pairs. The score was recorded in tenths of a second using a stopwatch.

**Obstacle course backward**. Participants had to walk backward on all four limbs for the distance of 10 m, climb to the top of a Swedish bench (30 cm), and crawl through the frame. The scores were recorded in tenths of a second using a stopwatch.

**Standing broad jump**. Participants had to jump with both feet from the reverse side of a Reuter bounce board onto a carpet of marked length. The score was the length of the jump recorded in cm.

**Arm plate tapping**. Participants had to alternately tap two plates on a tapping board for 15 sec with their dominant hand while holding the other hand in between the two plates. The number of double hits was recorded.

**Seated straddle stretch.** The subjects sat on the floor in a straddle position, leaning against the wall, and reached forward as far as possible along a straight-angle ruler. The score was the maximal reach measured in cm.

**Bent-arm hang**. Participants were instructed to hang in the pull-up position as long as possible (chin above the bar), holding the bar with an under-grip. The score was the time of the hold recorded in tenths of a second.

**Crossed-arm sit-ups**. Participants had to lie down on their back with knees bent and arms crossed on the opposite shoulders. They were instructed to rise into a seated position and return back to the starting position while the instructor’s assistant held the participants’ feet. The maximal number of correctly performed repetitions within 60 s was recorded.

Prior to the assessments, each test was demonstrated by the examiners. Subjects were allowed to perform a trial attempt for each test conducted.

### 2.6. Raven’s Coloured Progressive Matrices—Raven’s CPM

Raven’s Colored Progressive Matrices (CPM) represents a nonverbal test of intellectual abilities and discursive thinking, composed of perceptual, figurative material organized in several series, in which the tasks were arranged according to difficulty, from simple to complex [34]. The examination is performed visually. Nonverbal abilities that are assessed include (a) the ability to understand the complex relationships between stimuli, (b) the ability to find meaning among stimuli, and (c) abilities of perception and thinking. Within Raven’s CPMs, Set A is based on complementary continuous structure and is associated with visual–perceptual abilities. Set B requires the discovery of an analogy between elements. Test AB was initiated to reduce the transition in the opinion directorate. Raven’s CPM is a simplified form of the test, with 36 tasks divided into three series, with 12 tasks each. The background for drawings is painted to facilitate the maintenance of the participants’ attention. The items are arranged to assess cognitive processes, usually for children under 11 years of age or older and mentally impaired people.

The children were tested individually by trained psychologists in a quiet room of their schools. The instructions for applying the test were explained to participants in detail to facilitate application. A group of drawings that made up one whole or one principle was presented, with one of the drawings missing. The participant’s task was to discover specific relationships between the figures shown in the picture in a group of six drawings. The participant had to choose one of the six offered figures that fitted, according to a certain relationship. The number of correctly identified drawings was taken for further analysis [34]. A total of 132 children completed this test.

### 2.7. Cognitive Assessment System—CAS

The Cognitive Assessment System (CAS) [35] was administered in combination with eight selected subtests representing four different assessment scales. The matching numbers and planned codes subtests represented the planning scale. The simultaneous scale consisted of nonverbal matrices and verbal–spatial relations subtests. Expressive attention and number detection represented the subtests of the attention scale. The successive scale contained word series and sentence repetition [35,36].

A CAS battery test was conducted for each subject individually. Testing, using the basic form with eight CAS battery subtests, took an average of about 40 min. The time for solving the tasks, as well as the number of correct answers, was entered in the results form, where all subtests were later scored, and the subscale and total score on the test were determined.

Calculating raw scores differed depending on the subtest and involved the application of a certain method for recording the number of correct answers, incorrect answers, and the reaction time. Due to its complexity, the CAS assessment was completed only in a subset of 62 participants (intervention group = 23; control group = 39) at the baseline and follow-up.

### 2.8. Data Analysis

The independent-samples *t*-test and the chi-square test were used to compare demographic characteristics between the two groups. Furthermore, 2 × 2 repeated-measures ANOVA was used to compare differences in outcome measures after the intervention period between the two study groups, and the paired-samples *t*-test was used for calculating differences within subsamples. Moreover, *p*-values of <0.05 were considered statistically significant. No corrections were applied for multiple comparisons. The data are reported as mean ± SD or frequency (percentage). The data were analyzed using the statistical package SPSS version 20.0 (SPSS Inc., Armonk, NY, USA).

## 3. Results

The sample consisted of a total of 132 children aged 5.5 ± 0.7 years, with a mean height of 116.09 ± 6.37 cm and a mean weight of 21.71 ± 4.27 kg. Although no statistically significant differences were observed between the intervention and control groups at baseline, a greater proportion of children in the intervention group were boys and were either in the underweight or normal weight category compared to those in the control group (Table 2).

Table 3 presents data on children’s anthropometric characteristics and motor skills before and after the intervention in each group. Body weight increased significantly in both groups during the 9-month period, with a greater increase observed in the control group versus the intervention group. The control group also increased the waist circumference during the 9-month period, whereas no change was observed in the intervention group. Chest circumference increased in the intervention group, while no change was observed in the control group. No significant changes in forearm circumference, abdominal, subscapular and triceps skinfolds were observed following the 9-month period in either group.

The results of motor skills tests indicate certain changes among participants. Improvement in the *20-m dash* test has been noted in favor of the intervention group (*p* = 0.016). A significant difference was detected in the *standing broad jump* test (*p* = 0.000). This was particularly noticeable among the intervention group, considering that their mean values at baseline and postmeasurement were 108.09 ± 24.70 vs. 121.98 ± 20.44 cm, respectively. Intervention group subjects maintained their flexibility level, whilst the control group reduced their flexibility over time (*p* = 0.010), which can be noted in the *seated straddle stretch* test results. Statistically, a significant change was observed in the *bent-arm hang* test (*p* = 0.000) due to the improvement of the intervention group and impairment for the control group. Nevertheless, *obstacle course backward* (agility), *arm plate tapping* (simple movements), and *crossed-arm sit-ups* (repetitive core strength) remained stable over time.

The results on Raven’s CPM (cognitive skills test) significantly improved from preintervention to postintervention in both groups (Table 4). The results obtained using the CAS battery of tests indicated statistically significant changes between the intervention and control groups, which may be the result of the effects of the PA intervention or, more precisely, the result of the group–time interaction (Table 4). Statistically significant differences were noted in two variables: verbal–spatial relations and expressive-attention (*p* = 0.03 and *p* = 0.04, respectively). In verbal–spatial relations, the results demonstrated that significant changes occurred in favor of the control group, while the change in expressive attention was in favor of the intervention group.

## 4. Discussion

This study evaluated the effectiveness of a 9-month PA intervention on anthropometric characteristics, motor skills, and cognitive skills in preschool children. Significant improvements in running speed, jump distance, upper body strength, and flexibility were observed in the intervention group when compared to the control group. Both groups showed improvements in several cognitive test results, although each group demonstrated this improvement in a different subtest of variables, showing no robust evidence of PA-intervention-related improvements. While body weight increased in both groups during the 9-month period, the increase in body weight was greater in the control group versus the intervention group. Taken together, findings from this study suggest that regular PA contributes to the development of motor skills of preschoolers, while its effects on cognitive function remain inconclusive.

Only a paucity of studies to date has examined the effects of exercise training on physiological characteristics and motor and cognitive skills in preschool children [27,37,38]. Those studies have reported that both short-term (10- and 18-week interventions) and long-term (11-month intervention) exercise training led to an improvement in motor skills [27,37,38]. One study [27] found improvement in cognitive function (specifically, attentiveness) as a result of a 10-week PA intervention in young children. Findings from the present study add further evidence of the benefits of long-term PA intervention in improving the motor skills in preschool children, whereas there was no robust evidence that the PA intervention of a 60-min session, twice per week, resulted in improvement in cognitive function in this age group. Future studies with a larger sample size, randomized control design, and varied volumes of PA should examine whether PA interventions have effects on cognitive function in preschool children.

Given that insufficient PA in children is a global health problem, PA intervention studies such as this one can be a useful tool to further emphasize benefits that can be obtained through regular PA. Results from such studies could serve to pinpoint both the problem and potential strategies for informing the future development of curricula for preschool institutions.

However, a recent meta-analysis on the effectiveness of PA interventions in children who are 0 to 5 years old showed a small but statistically significant positive effect for interventions targeting increases in children’s moderate-to-vigorous PA, while no evidence of effect was observed for changing their light PA [39]. Even though it is hard to categorically explain why stronger effects are observed for moderate-to-vigorous PA, a plausible explanation would be the fact that most interventions to date have focused on implementing interventions with physical activities that are of an intensity that would modify levels of moderate-to-vigorous PA, rather than light PA or total PA [18]. In addition, another credible explanation for these findings would be due to light PA often being viewed as the secondary outcome measure, granted that, currently, there is more evidence for the health benefits of moderate-to-vigorous PA compared with light PA [40].

The strengths of this study include a 100% completion rate of study assessments (with the exception of the CAS test), a long PA intervention period (9 months), and trained staff involved in the delivery of the intervention and research data collection.

This study has several limitations. Small sample size and nonrandom assignment of participants to intervention and control groups represent research design limitations. In addition, the CAS assessment was completed only in a subset of participants. Moreover, although physiological changes observed in the intervention group were significant, it remains uncertain to what degree the PA intervention contributed to those changes and to what degree such changes were a result of normal growth and maturation. Finally, the effects of the PA intervention in this study could have been confounded by contextual variables such as socioeconomic constraints, lifestyle, and other institutional rules and practices that were not measured or controlled in the present study.

## 5. Conclusions

This study provides evidence that a 9-month PA intervention has beneficial effects on preschool children, demonstrating a positive influence on their motor and cognitive skills. Future studies with a larger sample size and randomized control design should examine whether PA interventions have effects on cognitive function in preschool children.

## Figures and Tables

**Table 1:** Activity plan for the intervention group.

Activity	Sep	Oct	Nov	Dec	Jan	Feb	Mar	Apr	May
Athletics	x							x	x
Sports games ^1^	x	x	x	x	x	x	x	x	x
Gymnastics	x	x	x	x	x	x	x	x	x
Yoga	x	x		x		x	x		
Outdoor activities	x							x	x
Aerobic		x			x	x	x		
Martial arts			x	x	x	x	x	x	
Dance			x						

^1^ Sports games included football (soccer), basketball, volleyball, team handball, and tennis.

**Table 2:** Demographic characteristics.

Cohort	Intervention Group (*n* = 66)	Control Group (*n* = 66)	*p*-Value
Age (years) (mean ± SD)	5.4 ± 0.8	5.6 ± 0.6	0.158
Age group [*n* (%)]			
4.0–4.9 years	22 (33.3%)	14 (21.2%)	0.271
5.0–5.9 years	27 (40.9%)	34 (51.5%)	
6.0–7.0 years	17 (25.8%)	18 (27.3%)	
Gender [*n* (%)]			
Boys	40 (60.6%)	32 (48.5%)	0.162
Girls	26 (39.4%)	34 (51.5%)	
Underweight and normal	60 (90.9%)	53 (80.3%)	0.201
Overweight	4 (6.1%)	10 (15.2%)	
Obesity	2 (3%)	3 (4.5%)	

**Table 3:** Effects of 9-month exercise program on anthropometric characteristics and motor skills in preschool children.

Variable	Intervention Group (*n* = 66)		*p*-Value within Group between Time	Control Group (*n* = 66)		*p*-Value within Group between Time	*p*-Value for Group by Time Interaction	η^2^
	Pre	Post		Pre	Post			
Anthropometric characteristics								
Height (cm)	115.60 ± 6.80	118.57 ± 6.90	<0.001	116.59 ± 5.95	119.77 ± 6.15	<0.001	0.431	0.01
Weight (kg)	21.27 ± 4.08	22.46 ± 4.41	<0.001	22.16 ± 4.47	23.79 ± 4.79	<0.001	<0.001	0.10
Chest circumference (cm)	56.96 ± 4.03	58.48 ± 4.07	<0.001	58.22 ± 4.82	58.55 ± 6.14	0.490	0.022	0.04
Waist circumference (cm)	55.06 ± 4.69	55.07 ± 5.04	0.966	54.36 ± 5.86	56.77 ± 6.35	<0.001	0.000	0.27
Forearm circumference (cm)	16.88 ± 1.35	17.30 ± 1.39	<0.001	17.31 ± 1.43	17.76 ± 1.46	<0.001	0.740	0.00
Abdominal skinfold (mm)	5.82 ± 2.63	6.13 ± 3.51	0.065	7.70 ± 4.85	7.72 ± 4.64	0.911	0.247	0.01
Subscapular skinfold (mm)	5.13 ± 1.60	5.29 ± 1.86	0.121	6.03 ± 2.67	6.26 ± 3.07	0.076	0.613	0.00
Triceps skinfold (mm)	8.57 ± 2.40	9.14 ± 3.20	0.002	9.51 ± 3.36	10.19 ± 3.69	<0.001	0.634	0.00
Motor skills								
20-m dash (s)	5.55 ± 0.83	5.27 ± 0.66	<0.001	5.57 ± 0.61	5.49 ± 0.54	0.053	0.016	0.04
Obstacle course backward (s)	26.18 ± 8.23	21.52 ± 6.98	<0.001	26.75 ± 9.66	23.53 ± 7.16	<0.001	0.117	0.02
Standing broad jump (cm)	108.09 ± 24.70	121.98 ± 20.44	<0.001	111.21 ± 19.33	112.98 ± 19.06	0.258	<0.001	0.16
Arm plate tapping (*n*/15 s)	14.95 ± 2.98	17.88 ± 2.99	<0.001	15.41 ± 2.94	18.50 ± 3.79	<0.001	0.708	0.00
Seated straddle stretch (cm)	37.53 ± 6.80	37.30 ± 7.82	0.722	37.03 ± 8.27	34.36 ± 8.38	<0.001	0.010	0.05
Bent-arm hang (s)	7.03 ± 7.70	13.18 ± 12.83	<0.001	11.42 ± 9.30	7.42 ± 6.61	<0.001	<0.001	0.21
Crossed-arm sit-ups (*n*/min)	16.32 ± 8.29	20.89 ± 9.50	<0.001	15.56 ± 8.80	20.09 ± 8.59	<0.001	0.977	0.00

Data are presented as means ± SD.

**Table 4:** Cognitive skills assessment.

Variable	Intervention Group		*p*-Value within Group between Time	Control Group		*p*-Value within Group between Time	*p*-Value	η^2^
	Pre	Post		Pre	Post			
Cognitive skills	(*n* = 66)			(*n* = 66)				
Raven’s Progressive Matrices in Colour (out of 36)	19.88 ± 5.16	23.02 ± 6.14	<0.001	19.45 ± 5.58	23.04 ± 5.66	<0.001	0.779	0.00
Cognitive Assessment System (CAS)	(*n* = 23)			(*n* = 39)				
Matching Numbers	8.22 ± 2.48	8.83 ± 3.12	0.252	6.97 ± 2.03	8.08 ± 2.38	0.003	0.419	0.01
Planned Codes	19.26 ± 10.93	21.22 ± 9.78	0.300	14.97 ± 8.87	16.15 ± 9.01	0.528	0.782	0.00
Nonverbal Matrices	12.61 ± 4.65	13.00 ± 3.17	0.718	10.10 ± 3.14	12.05 ± 3.65	0.001	0.156	0.03
Verbal–Spatial Relations	14.39 ± 2.35	13.91 ± 2.72	0.373	12.74 ± 2.96	13.97 ± 2.18	0.016	0.027	0.08
Expressive Attention	39.26 ± 13.40	45.35 ± 11.60	0.049	38.92 ± 11.33	41.28 ± 9.93	0.179	0.044	0.02
Number Detection	26.17 ± 9.36	29.78 ± 11.17	0.065	21.38 ± 6.52	26.49 ± 9.21	<0.001	0.505	0.01
Word Series	9.91 ± 2.50	10.17 ± 3.04	0.534	9.87 ± 2.75	10.13 ± 2.23	0.433	0.993	0.00
Sentence Repetition	5.83 ± 1.58	6.48 ± 2.10	0.155	6.03 ± 2.30	6.77 ± 2.35	0.006	0.848	0.00

Data are presented as means ± SD.
